# The Effects of Omega-3 Fatty Acids and Vitamin D Supplementation on the Nutritional Status of Women with Breast Cancer in Palestine: An Open-Label Randomized Controlled Trial

**DOI:** 10.3390/nu16223960

**Published:** 2024-11-20

**Authors:** Heba F. Almassri, Azidah Abdul Kadir, Mohammed Srour, Leng Huat Foo

**Affiliations:** 1 School of Health Sciences, Health Campus, Universiti Sains Malaysia, Kubang Kerian, Kota Bharu 16150, Malaysia; hebamassri204@gmail.com; 2 School of Medical Sciences, Health Campus, Universiti Sains Malaysia, Kubang Kerian, Kota Bharu 16150, Malaysia; azidahkb@usm.my; 3 Faculty of Medicine and Health Sciences, University of Palestine, Gaza Strip 890, Palestine; m.srour@up.edu.ps

**Keywords:** breast cancer, nutritional status, anthropometric measurements, blood albumin, dietary intake, omega-3 fatty acids, vitamin D

## Abstract

Background: This study emphasizes the critical role of early nutritional interventions in addressing cancer-related malnutrition. It aimed to assess the effects of omega-3 fatty acids (ω3) and vitamin D3 (VitD) supplementation on the nutritional status of newly diagnosed women with breast cancer (BC) in the Gaza Strip, Palestine. Method: A total of 88 newly diagnosed women with BC were randomly assigned into four groups: (i) Omega-3 fatty acid (ω3) group; (ii) Vitamin D (VitD) group; (iii) ω3+VitD group; and (iv) the controls. The patients took two daily 300 mg ω3 capsules and/or one weekly 50,000 IU VitD tablet for nine weeks. Nutritional status of the participants was assessed by several measurement tools, namely, the Patient-Generated Subjective Global Assessment (PG-SGA)-derived scores, anthropometric measurements, blood albumin status and dietary intakes between the baseline and after 9 weeks post-intervention. The procedures of the present study were registered on ClinicalTrial.gov with the identifier NCT05331807. Results: At the end of trial, there was a significant increase in the PG-SGA-derived nutritional risk scores (*p* < 0.01), body weight and body mass index (BMI) (both *p* < 0.05) among participants in ω3+VitD group compared to other groups. Additionally, there was a significant rise in blood albumin levels (*p* < 0.05), daily energy and protein intake in the ω3+VitD group (*p* < 0.05) compared to baseline. Conclusion: Participants with supplementation of daily ω3 and weekly VitD had improved nutritional status, assessed by the PG-SGA scores and anthropometric measures, blood albumin and dietary energy and protein intake among women with BC who were undergoing active treatment.

## 1. Introduction

Breast cancer (BC) is the most commonly diagnosed cancer among women, accounting for up to 6.9% of cancer deaths globally [1]. Occurrence of BC among women has surpassed other types of cancers as the most common cancer (30%), followed by lung and bronchus (13%), colon and rectum (8%), uterine corpus (7%), and melanoma of the skin (4%) [2]. In Palestine, approximately 5455 individuals were diagnosed with cancer in 2022, with BC comprising 15.8% of cases in the West Bank and 19.2% in the Gaza Strip [3].

It is well-documented that cancer and its treatments exert a wide range of adverse health effects, including malnutrition and treatment-related side effects that can significantly lower nutrient intake and cause unintentional weight loss [4]. There are several potential underlying causes associated with malnutrition risk among cancer patients such as agents produced by the tumor directly and/or systematically in response to the tumor such as pro-inflammatory cytokines and hormones, and chemotherapeutic agents with side effects such as nausea, vomiting, stomatitis, constipation and malabsorption have been implicated in the pathogenesis of malnutrition and cachexia [4]. Alterations in nutrient metabolism and resting energy expenditure could also be attributed to nutritional status [5]. Collectively, these factors can significantly hinder treatment response, heighten susceptibility to treatment-related adverse effects, and contribute to poor prognosis and quality of life outcomes [6,7].

An unintentional weight loss is primarily common among individuals with cancer, varying with tumor type and stage of progression [8]. The rapid weight loss in patients with cancer is associated with poor health outcomes such as reduced response to therapy, increased complications and infections, worsening of quality of life, and decreased survival [9]. Severe weight loss and malnutrition in cancer cachexia often leads to discontinuation of cancer treatment followed by decreased cancer survival [10]. Therefore, effective nutrition screening and early nutrition intervention, such as supplementation, are essential and should be implemented as nutritional support strategies to reduce the risk of malnutrition [11,12]. Incorporating these interventions into treatment programs can minimize the risk of malnutrition and its adverse effects, ultimately improving treatment outcomes [12]. A growing body of evidence has suggested that supplementation of specific nutrients such as an omega-3 fatty acid (ω3) has improved the nutritional status among patients with advanced cancer and/or those undergoing anticancer treatment [5,13], by reversing the metabolic catabolism found in most patients with cancer cachexia, improving appetite and body weight. Vitamin D (VitD) is also a nutrient of concern for cancer patients. For instance, VitD deficiency is highly prevalent among BC patients, especially at the time of disease diagnosis [14,15] and even after neoadjuvant chemotherapy [16]. Moreover, a significant inverse association was found between blood VitD levels and BC mortality [17], suggesting that optimal VitD status in the body is significantly associated with better disease prognosis status in BC patients. Numerous mechanistic studies have reported that VitD could inhibit the growth of tumor cells directly by regulating various genes responsible for cell proliferation, differentiation and apoptosis [18], as well as indirectly through regulating immune cells associated with the microenvironment of malignant tumor [18].

Most studies have documented the effects of individual ω3 or VitD on clinical outcomes among cancer patients [19,20], but research on the combined effects of ω3 and VitD supplementation on cancer patients is still lacking with only limited studies having been conducted so far on cancer risk or cancer patients [21,22]. Some showed favorable clinical outcomes such as improving nutritional status and inflammation [21], overcoming or delaying the development of resistance chemotherapeutic agents and reducing the side effects induced by the chemotherapy [23]. This could possibly be attributed to the synergetic effects of these nutrients on beneficial clinical outcomes in cancer patients.

To the best of our knowledge, there is little information pertaining to the effects of combining both ω3 and VitD supplementation on nutritional status and clinical outcomes of BC patients, particularly among those newly diagnosed. Additionally, there is little guidance on whether early nutritional support should be provided to these patients to mitigate malnutrition risk. This gap is especially pronounced in the middle-income regions, such as in Palestine, where healthcare access is restricted by economic, geographic, and cultural barriers [24], and many face challenges in obtaining a balanced diet. Micronutrient deficiencies can have significant health implications [25], underscoring the importance of timely nutritional interventions. This study aimed to assess the effects of ω3 and/or VitD supplementation on the nutritional status, assessed by various approaches, namely, Patient-Generated Subjective Global Assessment (PG-SGA) scores, anthropometric assessment, and blood albumin levels in patients of newly diagnosed BC in the Gaza Strip, Palestine.

## 2. Methods and Materials

### 2.1. Study Design

The present study was an open-labeled, randomized (1:1:1:1), controlled trial of women newly diagnosed with BC. Participants were selected and recruited from the Outpatient Clinic at the Turkish Palestinian Friendship Hospital in the Gaza Strip prior to the first chemotherapy treatment. The study protocols were approved by the Human Ethics Committee of the Universiti Sains Malaysia (USM) (Approval Code: USM/JEPeM/21090645) and conformed to the Declaration of Helsinki (Approval ID: PHRC/HC/943/21). The detailed procedures of the present study were registered in the ClinicalTrial.gov (Identifier No.: NCT05331807). In addition, a written informed consent was obtained prior to study screening for eligibility.

### 2.2. Selection of Participants in the Study

Eligible participants were selected and recruited from the hospital’s outpatient clinic, based on a list of BC patients diagnosed by medical oncologists and referred to the daily care unit prior to the medical chemotherapy treatment. Participants were screened using specific inclusion and exclusion criteria. The inclusion criteria were as follows: newly diagnosed with BC stages II or III, aged 28–64 years, without distant organ metastasis, and scheduled to receive their first chemotherapy treatment with Adriamycin + Cytoxan (AC) across four cycles (one cycle every 3 weeks, approximately 21 days each) with following diagnosis criteria: lymph node positive *+ve.*, hormonal receptor negative *-ve.*, and human epidermal growth factor receptor 2 negative [HER2: *-ve.*], normal blood biochemical tests of leukocyte and platelet counts of more than 3500 cells/mm^3^ and 100,000 cells/mm^3^, respectively. Exclusion criteria included the presence of other chronic conditions such as osteoporosis, renal disease, HIV, malabsorption disorders, autoimmune diseases, diabetes, hypertension, liver, parathyroid, or gastrointestinal disorders. Participants were also excluded if they had received other forms of oncology treatments (such as hormone or radiation therapy) instead of chemotherapy, had recurrent BC, a history of other cancer types, were currently taking ω3 or VitD supplements, receiving parenteral nutrition, had allergies to fish or seafood, or were pregnant.

### 2.3. Intervention and Randomization

The randomization numbers were conducted using a computer-generated randomization code based on a block size of eight that provided allocation of participant numbers in a ratio of 1:1:1:1 as, (i). group 1: omega-3 supplementation (ω3); (ii). group 2: vitamin D supplementation (VitD); (iii). group 3: both omega-3 and vitamin D supplementation (ω3+VitD); and, (iv). control group. The Sealed Envelope^TM^ method was employed for randomization, in which each code was sealed in opaque envelopes and numbered sequentially. A nurse who was not directly involved in the study was asked to open each envelope sequentially to randomize the patients in the study. Participants in the ω3 and/or VitD group received two daily 300 mg ω3 soft gel capsules and one weekly 50,000 IU VitD tablet, respectively, whereas participants in the control group did not receive any supplements and followed the usual treatment procedures without taking a placebo, due to difficulties in manufacturing a placebo capsule identical to the ω3 capsule. Each ω3 capsule (Omega 3 complex, Jamieson Laboratories, Windsor, ON, Canada) contained 180 mg Eicosapentaenoic acid (EPA) and 120 mg Docosahexaenoic acid (DHA), while VitD tablets (J-Dee) were manufactured and supplied by the Jerusalem Pharmaceutical Company, West Bank, Palestine and contained 50,000 IU vitamin D_3_. The patients were monitored regularly by the first researcher (HA) through regular phone calls and messages to ensure compliance. Compliance was evaluated by the total capsule count every week during the patients’ hospital visits through a meticulous count of the tablets. All data were collected from participants at baseline (2 weeks after first chemotherapy session (T_baseline_) and at the end of the completed trial at 9 weeks as end-trial (T_end-trial_)). In addition, safety assessments included adverse events that occurred during the reporting period.

### 2.4. Sample Size Calculation

The sample size was determined using PS: Power and Sample Size Calculation software Version 3.0 for comparing two means. Sample size estimation was performed for all outcome measures used, and the largest required sample size was selected. This estimation assumed that the largest difference would be observed in nutritional status, assessed by the PG-SGA between supplemented and the control groups, conducted in a previous study by Qiu and co-workers in 2020 [26], using a fixed factor levels model (determining sample size for analysis of variance) and sigma value of 2.717 with 90% power, and α of 0.05. A total of 22 participants were required for each group. To allow for dropping out, 24 patients were recruited for each group. Participants in each experimental group were matched and stratified by age group (±5 years), menopausal status, disease stage, and BMI (±2.0 kg/m^2^). At the end of the study, a total of 96 patients with stage ӀӀ or ӀӀӀ BC cancer were included in the final analysis.

### 2.5. Data Collection and Outcome Measures

Data on histopathological diagnosis, cancer stage, and treatment were obtained from the medical records. The primary outcome was nutritional status assessed by PG-SGA and anthropometric measurements, while the secondary outcome was dietary intake and blood albumin levels. Various study procedures were employed, including face-to-face interviews, anthropometric measurements, and blood collection.

### 2.6. General Socio-Demographic Characteristics and Anthropometric Assessments

A face-to-face interview was conducted based on a structured questionnaire to assess the socio-economic and demographic profile, dietary patterns, and lifestyle-related behavioral practices such as daily intakes of breakfast, fruits and vegetables, cooking methods, special diet practice and smoking habits. Habitual physical activity level was assessed based on a short version of the International Physical Activity Questionnaire (IPAQ-S), which consists of 7 questions in a week time dimension [27]. In addition, information on personal medical history such as family cancer history, relatives affected by BC, disease stage, diagnosis date, and surgical treatment type were also gathered.

Body weight was measured to an accuracy of 0.1 kg using a weighing scale (Model: SECA 876, Hamburg, Germany), while height was assessed to the nearest 0.5 cm in the standing position without shoes by using a stadiometer (Model: SECA 201, Hamburg, Germany). BMI was expressed as the weight (kg) divided by height (m^2^). Waist circumference was measured with a non-stretchable tape measure, recorded to the nearest 0.1 cm. The measurement was taken horizontally around the abdomen at the level of the landmarked point, drawn at the uppermost lateral border of the iliac crest. For calf circumference, a non-stretchable measuring tape was looped horizontally around the calf. The tape was moved up and down until the greatest calf circumference was found, and the circumference was recorded to the nearest 0.1 cm. The evaluation of treatment safety was based on adverse events reported by the participants, and biochemical parameters (urea and creatinine) were measured at the baseline and at the end of the trial during the hospital visits.

### 2.7. Dietary Intake Assessment

Dietary nutrient intakes of these participants were assessed using non-consecutive three days of past 24 h dietary recalls, comprising two weekdays and one weekend at the beginning of the study and nine weeks of the intervention. The participants were requested to recall all foods and beverages within the past 24 h, including portion size, cooking methods, brand name, time, and venue of each food taken. The participants were instructed not to alter their regular dietary habits. Standard household measuring cups, glasses, bowls, and spoons were also used to assist participants to estimate their meal portion sizes. Dietary analyses using the Nutritionist Pro™ software (Axxya Systems LLC, version 8.01, Redmond, WA, USA) were used to analyze the nutrient profiles for foods and beverages, based on the USDA Standard Reference Nutrient Database. When any of the databases did not have nutrient information for specific food items, detailed information of local traditional meals such as meal recipes, raw and packaged ingredients used (such as specific food brands) and quantities used to prepare the meal were gathered. A standard recipe was then included and analyzed using the Nutritionist Pro™ software.

### 2.8. Assessment of PG-SGA-Derived Nutritional Status Risk

The PG-SGA was utilized to assess the nutritional status risk of BC patients at the beginning and at the end of the nine weeks’ intervention trial. The PG-SGA stands as a reliable and valid tool, offering a reference point for identifying and categorizing nutritional risk conditions in cancer patients [28]. The first section of the PG-SGA questionnaire was completed by the patients and consisted of an evaluation of weight loss history at six months and one month prior to the interview. Section two of the PG-SGA focuses on data that were obtained during the visit with the clinician, such as diagnosis, age, physical examination, and metabolic stress [29]. Each section was then summed into a total score of nutritional risk, with a greater score representing a higher nutritional risk status. The general score ranges from 1 to 40. A total score of 9 or more represents a critical need for nutritional intervention and a dietary consultation for the respective patients [30]. The patients were then classified as well nourished, moderately malnourished and severely malnourished.

### 2.9. Determination of Blood Biochemical Albumin Status

Blood samples were collected after 12 h overnight fasting by a trained medical laboratory technologist or staff nurses at the hospital at the study’s initiation and after nine weeks of intervention. Blood serum was collected by centrifugation at 800–1000 rpm for about 10 min. The serum was separated and stored at −80 °C immediately prior to analysis. The Bromocresol Green Method was used to measure the biochemical albumin levels in blood using (Quimica Clinica Aplicada S.A. Kit, Amposta, Spain). The reference value for albumin in the blood was set at 3.5 to 5.0 g/dL.

### 2.10. Statistical Analysis

The normal distribution of variables was assessed and determined by the Kolmogorov–Smirnov test. If the *p*-value of the Kolmogorov–Smirnov test is larger than 0.05, then the data have a normal distribution, and parametric statistical analysis, specifically, a one-way analysis of variance (ANOVA), was conducted. Conversely, if the data do not follow a normal distribution (*p* < 0.05), non-parametric statistical analyses, such as the chi-square test, were performed. Quantitative parameters were presented as mean ± standard deviation (SD), while categorical variables were shown as frequencies and proportions. The baseline comparisons of study variables between groups were compared using one-way analysis of variance (ANOVA). Comparison of variables within the groups from baseline (T_baseline_) and at the end of intervention (T_end-trial_) was performed using a paired sample *t*-tests for all continuous variables. The mean change in each outcome measure at the end of trial between these intervention groups was tested using the multivariate analysis (ANCOVA), after adjusting for baseline outcome value, income, age and the stage of BC. In addition, the Bonferroni test was used for pairwise comparisons. All statistical analyses were performed using the SPSS (version 27.0; SPSS Inc., Chicago, IL, USA) [31] with statistical significance for all tests defined by the *p*-value of <0.05.

## 3. Results

**Figure 1:**
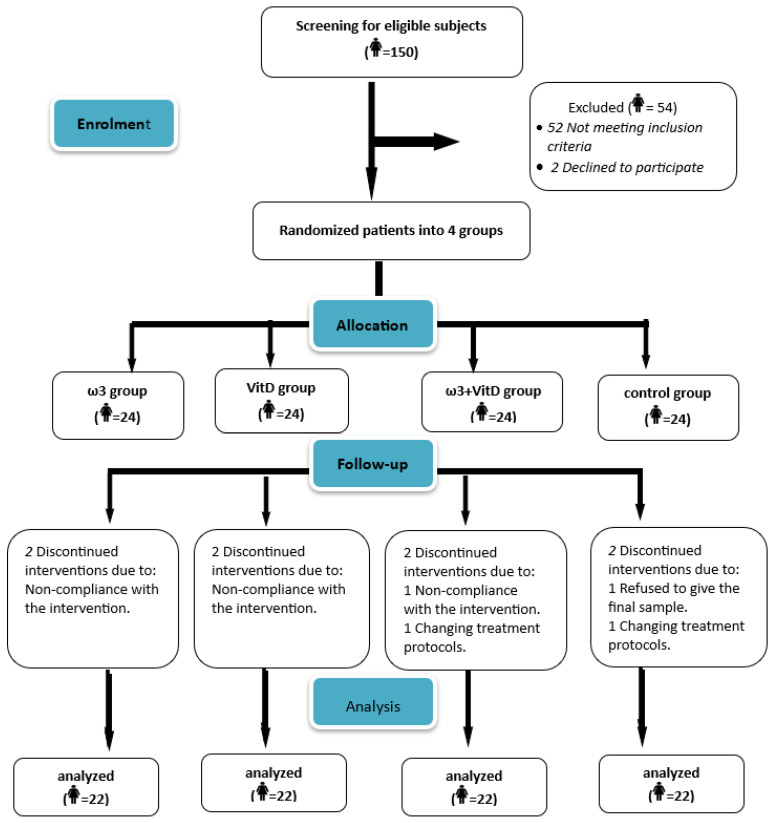
Flow diagram of the study. (i) Patients receiving ω3; (ii) patients receiving VitD; (iii) patients receiving both ω3 and VitD; and (iv) control as usual treatment group.

Figure 1 shows the trial enrolment based on the CONSORT flow diagram. About 150 women were screened in the present study, of which 54 of them were excluded based on the inclusion and/or exclusion criteria. A total of 96 eligible participants were recruited and included in the study. The recruitment of participants commenced on 18 May 2022, and extended with follow-ups until the end of December 2022. Throughout the study, eight (8) participants dropped out due to several reasons such as non-compliance with supplements, refusal to provide blood sample at the end of the study or changes in the treatment plan by the oncologist. Consequently, the final number included in the analyses was 88 participants.

### 3.1. General Characteristics of the Participants at Baseline

Table 1 shows the baseline information for socio-demographic, dietary and lifestyle characteristics of the participants based on the experimental groups. In general, there were no significant differences for most socio-demographic, dietary and lifestyle-related factors between these four groups. In addition, clinical characteristic parameters were assessed between the experimental groups.

### 3.2. Nutritional Risk Status Assessed by the PG-SGA, Anthropometry and Blood Albumin Levels of the Participants

Table 2 presents the nutritional risk status, assessed by the PG-SGA, anthropometry and blood albumin levels of the participants based on four experimental groups. At baseline, there were no significant differences for the PG-SGA-derived nutritional risk scores, all anthropometric measures and blood albumin levels. Comparisons within the group at the end of the trial over 9 weeks showed a significant increment in the PG-SGA score in the control group from the baseline, suggesting a deterioration of nutritional status (*p* = 0.005). On the contrary, participants in the ω3+VitD group had significantly reduced the scores of PG-SGA at the end of the trial (*p* = 0.009). Moreover, participants of ω3+VitD group had significant increases in the body weight and BMI levels (both *p* = 0.028), whereas a significant decrease in calf circumference was found in the control group when compared with the baseline measure (*p* = 0.021). Similarly, a significant increase in blood albumin level was found only in the ω3+VitD group (*p* = 0.042). When the outcome of measurements was compared between intervention groups, it was found that the scores of the PG-SGA were significantly different between the groups (*p* = 0.001). Participants in the ω3+VitD group had significantly lower PG-SGA scores (*p* < 0.001) compared to control group at the end of the trial. There were significant differences for body weight and BMI levels, whereby participants of ω3+VitD group had significantly higher BMI levels (*p* = 0.032) when compared with the control group. However, no significant differences were observed in serum albumin levels between these intervention groups.

### 3.3. Dietary Intakes of the Participants

Table 3 shows the daily nutrient profile of the participants according to the intervention groups. In the baseline, no significant differences were found for most nutrients such as daily intakes of energy, macronutrients, ω3 and VitD intakes. However, after 9 weeks of supplementation, a significant increase was observed in daily intakes of energy (*p* = 0.014), protein (*p* = 0.043), and fat (*p* = 0.031) among the participants in ω3+VitD group, and dietary energy and protein intakes in ω3 group (*p* = 0.039, and *p* = 0.043, respectively) from the baseline intakes. Moreover, comparisons between intervention groups found that participants of ω3+VitD group had significantly higher intakes of fat compared to other groups (*p* = 0.033).

### 3.4. Safety Measurements

Table 4 shows patient-reported adverse events throughout the study period. A total of 4 (4.5%) adverse events were recorded. All the adverse events that occurred in the ω3 and ω3+VitD groups were related to the intakes of the ω3 supplement, such as nausea and abdominal discomfort. Only one adverse event, dry mouth, occurred in the VitD group. Similarly, a safety assessment was conducted by blood biochemical markers of urea and creatinine at the baseline and at the end of the trial (Table 5). It shows that there were no significant differences for blood urea and creatinine levels between these four experimental groups.

## 4. Discussion

The main findings of the present study demonstrate that participants who had received both daily 600 mg ω3 and weekly 50,000 IU VitD supplements had significantly reduced the PG-SGA-derived nutritional risk scores and increased body weight and BMI during the active chemotherapy treatment for nine weeks. In addition, participants of ω3 and VitD co-supplementation group had significantly higher concentrations of albumin and higher daily intakes of energy and macronutrients.

To the best of our knowledge, this was a first study of its kind that was conducted using both ω3 and VitD supplementation with a nutrient supplementation design among women newly diagnosed with BC in Palestine. This distinction is significant as early nutritional interventions are not widespread in Palestine, a developing country grappling with limited resources and financial constraints, resulting in low purchasing power among its population. This study addresses a critical gap in existing healthcare practices, shedding light on the importance of tailored nutritional support in a context where such initiatives are not conventionally prioritized. In addition, assessing the effects of combined benefits of both nutrients of concern in BC participants in the present trial was a novel approach compared to individual use of these nutrients. It is hoped that the pioneering effort can contribute valuable insights that may pave the way for enhanced care strategies and more effective interventions to be used for women newly diagnosed with BC in the present populations and other similar resource-constrained settings globally. The findings of this study have far-reaching implications, emphasizing the need for innovative and accessible approaches to healthcare in regions facing financial challenges and limited resources. The findings of the present study of nutritional status assessed by anthropometry are consistent with a previous recent study conducted by Haidari and her co-workers among 81 colorectal cancer adults, whereby nutritional status that was assessed by body weight and BMI was significantly increased at the end of 8 weeks of intervention which involved supplementation with both daily dosage of 660 mg ω3 and weekly 50,000 IU of VitD [21].

At baseline, a significant portion of our BC patients were either overweight or obese, aligning with similar observations in other studies [32,33]. This is consistent with the recognition of obesity as a major risk factor for cancer, indicating the fact that obesity is one of the determinant factors associated with cancer development risk [34]. Anthropometric assessment serves as a valuable supplementary method for evaluating the risk of malnutrition in cancer patients [35]. The nutritional screening and assessment guidelines for cancer patients, as highlighted by the European Society of Parenteral and Enteral Nutrition (ESPEN), state that nutritional risk assessment that includes current nutritional intake, weight changes, and BMI starting from the moment of cancer diagnosis are particularly important in order to identify the nutritional disturbances during the early stage of cancer development, with periodic reassessments aligned with clinical stability [5,36]. Using this nutritional risk assessment tool, this study was able to identify the high risk of patients who experienced mild to moderate malnutrition, by taking into account factors that are beyond BMI assessment, such as reduced dietary intake, muscle loss, and weight loss [37]. In the present study, it was found that participants in both ω3 and VitD group had significantly greater body weight and BMI levels compared to other groups. Interestingly, participants of both supplementations also reported a significant increase in daily consumption of energy, protein, and fat. The substantial enhancement in nutritional status, as reflected in body weight and BMI levels, observed in the ω3+VitD group might be linked to the elevated intake of energy, protein, and fat in their diet. These findings align with a previous study that highlighted a strong positive correlation between dietary energy and protein intake and weight changes among cancer patients [38]. Additionally, prolonged and significant weight loss (>5% or reduction in BMI or category change) following diagnosis has been linked to diminished long-term survival in cancer [39]. Furthermore, the results of Aredes and co-workers [13] indicated the importance of ω3 in weight maintenance among cancer patients undergoing treatments. In a recent investigation conducted by Cheng and his co-workers, participants who were given a 12-week supplementation of 1.6 g EPA and 0.8 g DHA also reported a significant higher body weight than the placebo group [40].

Moreover, co-supplementation with ω3 and VitD significantly reduced PG-SGA scores compared to the other intervention groups and the control. Numerous studies in cancer patients have focused on the uses of ω3 in several types of cancers showing a contradictory result. For instance, a study using 2.5 g daily ω3 for 45 days in patients with cervical cancer undergoing chemotherapy showed a favorable reduction in PG-SGA scores [13], whereas another study by Feijo and colleagues (2019) among gastric cancer patients showed no significant change in PG-SGA scores between experimental groups [41]. Similarly, no significant change was found in the PG-SGA score at the individual ω3 or VitD group in relation to the control in the present study. Additionally, the present study also demonstrated a significantly higher level of blood albumin among participants in the combined ω3 and VitD group after 9 weeks of supplementation, whereas neither ω3 nor VitD group showed significant changes in the circulating levels of albumin in the blood. It is generally agreed that blood albumin is a proxy indicator of total body protein status, where its turnover in the body is highly influenced by the disease state and current dietary intakes [42]. Hence, low albumin levels in the blood have been regarded as a proxy indicator of malnutrition risk [43]. In the present study, a significant increase in blood albumin levels among the participants of combined ω3+VitD supplements over 9 weeks is in accordance with the observation of a clinical trial study of 81 colorectal cancer patients [21]. In the latter study, supplementation of ω3 alone increased the blood albumin levels, suggesting that supplementation of ω3 and/or VitD supplements could help to improve blood albumin status. However, we were unable to find a significant change in blood albumin levels in either the ω3 or VitD (alone) groups.

In the present study, all participants had a low daily energy intake of <25 kcal per kg of their body weight, which was based on the ESPEN guidelines recommended levels for cancer patients [5]. However, after 9 weeks of receiving both ω3 and VitD supplements, there was a significantly higher intake of dietary energy, fat, protein, EPA, DHA and VitD compared to baseline. It is generally agreed that malnutrition is associated with lower tolerance to anticancer treatment and increases the risk of morbidity and mortality in cancer patients [35]. For instance, appetite loss induced by tumor-associated substances such as pro-inflammatory cytokines, lipid mobilization factor, and proteolysis triggering factor and severe weight loss in cancer patients are significantly associated with important clinical outcomes such as decreased prognosis and survival, fewer completed cycles of chemotherapy, more treatment side effects as well as reduced health-related quality of life [44]. Numerous studies of using different nutritional strategies have documented a significant improvement in weight gain that can be used as an adjuvant support to reduce the risk of malnutrition in cancer patients [45]. It has been found that co-supplementation of ω3 and VitD could produce favorable effects on their nutritional status by increasing body weight and BMI levels [21].

Numerous studies have documented that the uses of ω3 supplements in cancer patients could remarkably reduce the systemic inflammation levels [40], improve appetite and food intake, enhance lean body mass and weight gain [46,47] and cause reduction in the risk of nausea/vomiting, anorexia symptoms and fatigue [48]. On the other hand, the beneficial effects of VitD in cancer patients are well-documented, with studies showing that 90% of advanced cancer patients have a VitD deficiency. Research by Koole et al. (2020) and Martínez-Alonso et al. (2016) highlights the link between low VitD levels and increased fatigue [49,50]. Additionally, VitD supplementation has been associated with favorable clinical outcomes, including reduced pain, improved gastrointestinal symptoms (such as nausea and vomiting), and increased appetite [51]. These symptoms associated with VitD deficiency can lead to decreased dietary intake, consequently impacting nutritional status. Several recent studies have suggested that the positive outcomes observed with the combined use of ω3 and VitD may result from a synergistic effect [52,53]. There are several proposed mechanisms of action behind these effects that might stem from their distinct actions. For example, VitD regulates serotonin levels by influencing synthesis, while EPA regulates serotonin release by inhibiting E2 series prostaglandin production, and DHA modulates serotonin function by enhancing neuronal cell membrane fluidity [54]. The synergistic effects of ω3 and VitD on the serotonin system could lead to improved health and behavior [54]. Moreover, numerous studies have increasingly highlighted that ω3 supplementation can influence VitD levels in the body [52,55]. Research has shown that fish oil, a primary source of ω3, can increase serum VitD levels [55]. Additionally, a systematic review and meta-analysis of 10 randomized controlled trials demonstrated a significant increase in 25(OH)D levels with ω3 supplementation. Notably, 25(OH)D levels were markedly higher when the supplementation duration exceeded eight weeks, particularly when baseline serum 25(OH)D levels were below 20 ng/mL [53]. Furthermore, the research indicates that the administration of ω3 and VitD at this dosage is safe and well tolerated for individuals undergoing chemotherapy. The outcomes of our study are consistent with those reported in earlier published trials [56,57]. These observations from numerous previous clinical trial studies are in line and consistent with the current finding of participants who received ω3 and VitD supplements, in which supplementation of both nutrients considerably improved nutritional status. Hence, the usage of both supplementation seem to be applicable to the present Palestine populations, where the majority of populations consume less fish and seafood, which is a main source of ω3, and at the same time, where there is a high prevalence of VitD deficiency, as defined by the blood 25-hydoxyvitamin D level of <25 nmol/L, among female adults [58]. Overall, VitD plus ω3 supplementation for nine weeks in stage ӀӀ or ӀӀӀ BC patients undergoing chemotherapy has a strong synergistic impact on nutritional status assessed by anthropometry and blood albumin levels and PG-SGA scores, compared to other groups. A targeted nutritional approach, including the mentioned supplementation, could prove to be an effective strategy for BC therapy. We recommend confirmation of these findings be obtained by further, larger studies with a longer follow-up period. It is hoped that specific and effective nutritional intervention supports such as supplementation of both ω3 and VitD could be incorporated as adjuvant therapy to improve the nutritional status and reduce the nutritional risk among cancer patients during the active anticancer treatment period.

This study has some limitations. First, the study was not blinded and was conducted without placebo due to manufacturing difficulties in producing a placebo capsule that was identical to the ω3 capsule. Secondly, assessment of blood biochemicals of ω3 and VitD levels was not carried out to assess the blood nutritional status of ω3 and VitD due to absence of the kits and suitable equipment that are required to measure ω3 in Palestine and, also due to financial constraints. However, this does not weaken the main outcomes of the study mainly focused on nutritional status and nutritional risk status. These financial and logistical limitations highlight the need for better resource allocation and enhanced laboratory infrastructure in future studies. Limited funding and the lack of required equipment in local facilities prevented us from conducting comprehensive biochemical analyses, such as precise measurement of ω3 and VitD blood levels. To address these limitations, future research would benefit from targeted investment in laboratory resources and diagnostic tools, potentially achieved through partnerships with international organizations or regional health institutions. Such initiatives would facilitate more extensive data collection, enabling more accurate assessment of biochemical markers, thereby reinforcing study validity and supporting advancements in cancer care within resource-limited settings. Despite the limitations, the present study has number of strengths. It notably marked the first-of-its-kind investigation into the effects of individual ω3 and VitD and both nutrients on several important clinical outcomes, namely nutritional status and nutritional risk assessed in women newly diagnosed with BC, by both objective anthropometry measurement and subjective PG-SGA score assessments, respectively. Another significant strength is its nature as a randomized controlled study that integrates nutritional interventions with good (90%) adherence to the supplements. Lastly, the current randomized controlled trial was conducted by considering other factors such as dietary and lifestyle-related behavioral practices in Palestine, for which there is a lack of scientific evidence related to nutritional support, especially concerning early nutritional interventions. This gap could hinder the implementation of suitable and effective nutritional support for patients newly diagnosed with cancers. This is particularly significant in the context of Palestine being a developing country with limited intervention studies. Such studies are crucial, especially given the scarcity of comparable research conducted in regions with fewer resources. However, a larger clinical trial with a larger sample size is still needed to validate the positive impacts of combining ω3 and VitD in supporting BC patients.

## 5. Conclusions

The study findings indicate that BC patients receiving combined supplementation of 600 mg of daily ω3 and 50,000 IU weekly VitD over nine weeks experienced significant improvements in nutritional status, as measured by PG-SGA scores and anthropometric assessments, compared to the control group. Furthermore, increases in blood albumin levels and dietary intakes of energy and macronutrients were observed at the conclusion of the study, relative to baseline levels. These findings indicate that the combined ω3 and VitD supplementation may serve as a promising and effective adjuvant nutritional support for newly diagnosed cancer patients undergoing active treatment. Future studies should focus on optimizing the design of ω3 and VitD combinations. Moreover, larger-scale clinical trials with more diverse and representative populations, as well as extended durations, are crucial to assess key health outcomes in cancer patients, such as treatment adherence and overall survival rates. This will enhance our understanding of how this supplementation regimen impacts cancer-supportive care strategies for individuals undergoing active anticancer treatment.

## Figures and Tables

**Table 1:** Baseline general characteristics of the participants in the different supplementation groups.

Variables	ω3 (*n* = 22)	VitD (*n* = 22)	ω3+VitD (*n* = 22)	Control (*n* = 22)	*p*-Value
	*n* (%)				
Age (years) ^a^	45.4 (10.4)	47.3 (10.0)	47.2 (8.2)	47.3 (8.8)	0.879
Educational level					0.606
- Illiterate	0	1 (4.5)	0	1 (4.5)	
- Primary school	2 (9.1)	1 (4.5)	1 (4.5)	13.6 (3)	
- Middle to secondary schools	11 (50.0)	12 (54.5)	16 (72.7)	14 (63.6)	
- College or above	9 (40.9)	8 (36.4)	5 (22.7)	4 (18.2)	
Residential area					0.516
- Village	4 (18.2)	6 (27.3)	6 (27.3)	3 (13.6)	
- Camp	11 (50.0)	8 (36.4)	13 (59.1)	11 (50.0)	
- City	7 (31.8)	8 (36.4)	3 (13.6)	8 (36.4)	
Occupation status					0.635
- Employed	2 (9.1)	3 (13.6)	1 (4.5)	1 (4.5)	
- Housewife	20 (90.9)	19 (86.4)	21 (95.5)	21 (95.5)	
Marital status					0.331
- Single	1 (4.5)	3 (13.6)	0	0	
- Married	18 (81.8)	17 (77.3)	18 (81.8)	18 (81.8)	
- Divorce/Widowed	3 (13.6)	2 (9.1)	4 (18.2)	4 (18.2)	
Monthly income level ^b^					0.188
- Very low income	12 (54.5)	11 (50.0)	12 (54.5)	10 (45.5)	
- Low income	7 (31.8)	7 (31.8)	7 (31.8)	5 (22.7)	
- Middle income	3 (13.6)	4 (18.2)	0	6 (27.3)	
- Upper-middle and high income	0	0	3 (13.6)	1 (4.5)	
Dietary practices					
Daily breakfast intake status					0.109
- Yes	14 (63.6)	20 (90.9)	18 (81.8)	19 (86.4)	
Preferred cooking method					0.351
- Frying	8 (36.4)	8 (36.4)	13 (59.1)	6 (27.3)	
- Grilling	2 (9.1)	1 (4.5)	2 (9.1)	1 (4.5)	
- Boiling	12 (54.5)	13 (59.1)	7 (31.8)	15 (68.2)	
Daily vegetable intake status					0.300
- Yes	14 (63.6)	18 (81.8)	14 (63.6)	18 (81.8)	
Daily fruit intake status					0.086
- Yes	10 (45.5)	12 (54.5)	4 (18.2)	9 (40.9)	
Special diet practice					0.856
- None	20 (90.9)	19 (86.4)	19 (86.4)	18 (81.8)	
- Other (low carbs and sugar or low salt or weight reduction)	2 (9.1)	3 (13.6)	3 (13.6)	4 (18.2)	
Lifestyle practices					
Weekly physical activity status					1.000
- Low	16 (72.7)	16 (72.7)	16 (72.7)	17 (77.3)	
- Moderate	5 (22.7)	5 (22.7)	5 (22.7)	4 (18.2)	
- High	1 (4.5)	1 (4.5)	1 (4.5)	1 (4.5)	
Smoking habits					0.361
- Smoker	0	0	1 (4.5)	0	
- Passive smoker	11 (50.0)	10 (45.5)	5 (22.7)	8 (36.4)	
- Non-smoker	11 (50.0)	12 (54.5)	16 (72.7)	14 (63.6)	
Medical history					
Family history of cancer					0.571
- Yes	14 (63.6)	13 (59.1)	14 (63.6)	10 (45.5)	
Breast Cancer stage ^c^					1.000
- Stage II	12 (54.5)	12 (54.5)	12 (54.5)	12 (54.5)	
- Stage III	10 (45.5)	10 (45.5)	10 (45.5)	10 (45.5)	
Surgical treatment status					0.930
- No surgery	13 (59.1)	15 (68.2)	13 (59.1)	13 (59.1)	
- Lumpectomy	6 (27.3)	4 (18.2)	6 (27.3)	4 (18.2)	
- Mastectomy	3 (13.6)	3 (13.6)	3 (13.6)	5 (22.7)	

ω3 = omega-3 group; VD = vitamin D group; ω3+VD = vitamin D–omega3 supplementation group. Abbreviation: NIS, New Israel Shekel. ^a^ Values are presented in mean ± SD. ^b^ Income status was classified based on Palestinian Central Bureau of Statistic; Very low income (<1000 NIS), Low income (1001–1974 NIS), Middle income (1975–2470 NIS) Upper-middle and high income (>2470 NIS). ^c^ BC stage was defined based on the American Joint Committee on Cancer (AJCC) TNM system (2018).

**Table 2:** Comparison of nutritional status between baseline and end-line of the participants in the different supplementation groups.

Variables	ω3 (*n* = 22)	VitD (*n* = 22)	ω3+VitD (*n* = 22)	Control (*n* = 22)	*p*-Value
	Mean ± SD				
Total PG-SGA Scores					
- Baseline	7.2 ± 3.0	7.2 ± 2.7	6.8 ± 2.7	6.8 ± 2.5	0.920
- End of trial	6.8 ± 3.2	7.7 ± 2.3	5.8 ± 1.8	8.4 ± 3.1	
- Adjusted change	−0.03 ± 0.4 ^c^	0.3 ± 0.4 ^c^	−1.1 ± 0.4 ^c^	1.1 ± 0.3 ^c^	0.001 ^b^
- *p*-value ^a^	0.323	0.219	0.009	0.005	
Body weight (kg)					
- Baseline	77.7 ± 12.2	73.0 ± 12.8	77.1 ± 12.8	77.6 ± 10.5	0.502
- End of trial	78.3 ± 12.1	72.5 ± 12.6	78.4 ± 12.6	76.6 ± 10.0	
- Adjusted change	0.5 ± 0.4 ^c^	−0.4 ± 0.40 ^c^	0.7 ± 0.4 ^c^	−0.5 ± 0.4 ^c^	0.022 ^b^
- *p*-value ^a^	0.090	0.071	0.028	0.030	
BMI (kg/m^2^)					
- Baseline	30.6 ± 4.6	29.0 ± 4.5	30.5 ± 5.3	30.0 ± 3.8	0.611
- End of trial	30.8 ± 4.6	28.8 ± 4.4	31.0 ± 5.2	29.6 ± 3.4	
- Adjusted change	0.2 ± 0.1 ^c^	−0.1 ± 0.2 ^c^	0.3 ± 0.1 ^c^	−0.2 ± 0.1 ^c^	0.016 ^b^
- *p*-value ^a^	0.088	0.067	0.028	0.024	
Waist circumference (cm)					
- Baseline	99.0 ± 12.4	96.5 ± 12.5	101.7 ± 11.4	101.2 ± 10.6	0.436
- End of trial	99.0 ± 12.2	95.8 ± 12.5	102.4 ± 11.1	100.5 ± 10.0	
- Adjusted change	0.0 ± 0.3 ^c^	−0.6 ± 0.3 ^c^	0.3 ± 0.3 ^c^	−0.3 ± 0.3 ^c^	0.322 ^b^
- *p*-value ^a^	0.888	0.023	0.158	0.074	
Calf circumference (cm)					
- Baseline	39.6 ± 4.4	37.8 ± 4.0	39.9 ± 4.2	39.5 ± 4.4	0.353
- End of trial	39.7 ± 4.5	37.5 ± 3.8	40.0 ± 4.1	39.3 ± 4.5	
- Adjusted change	0.1 ± 0.1 ^c^	−0.2 ± 0.1 ^c^	0.0 ± 0.1 ^c^	−0.1 ± 0.1 ^c^	0.272 ^b^
- *p*-value ^a^	0.427	0.083	0.266	0.021	
Blood albumin (g/dL)					
- Baseline	4.24 ± 0.29	4.19 ± 0.28	4.17 ± 0.22	4.17 ± 0.34	0.826
- End of trial	4.26 ± 0.22	4.24 ± 0.30	4.35 ± 0.30	4.13 ± 0.27	
- Adjusted change	0.01 ± 0.07 ^c^	0.02 ± 0.07 ^c^	0.16 ± 0.07 ^c^	0.01 ± 0.06 ^c^	0.198 ^b^
- *p*-value ^a^	0.773	0.524	0.042	0.662	

ω3 = omega-3 group; VitD = vitamin D group; ω3+VitD = vitamin D–omega3 group. Abbreviation: PG-SGA, Patient-Generated Subjective Global Assessment. ^a^ Within-group differences; *p*-value is based on paired *t*-test. ^b^ Analysis was carried out by covariance (ANCOVA), after adjusting for baseline value, age, income and cancer stage. ^c^ Mean difference represented by adjusted mean change and standard error mean.

**Table 3:** Comparison of dietary intake between baseline and end-line of the participants in the different supplementation groups.

Variables	ω3 (*n* = 22)	VitD (*n* = 22)	ω3+VitD (*n* = 22)	Control (*n* = 22)	*p*-Value
	Mean ± SD				
Energy (Kcal/d)					
- Baseline	1344.1 ± 203.9	1516.7 ± 255.8	1350.6 ± 317.4	1370.8 ± 343.2	0.154
- End of trial	1521.2 ± 314.3	1508.5 ± 345.3	1545.9 ± 234.3	1316.1 ± 185.6	
- Adjusted change	139.5 ± 73.8 ^c^	7.3 ± 72.4 ^c^	145.3 ± 68.0 ^c^	23.0 ± 66.0 ^c^	0.319 ^b^
- *p*-value ^a^	0.027	0.920	0.014	0.490	
Energy (Kcal/kg)					
- Baseline	17.8 ± 4.0	21.5 ± 5.6	18.3 ± 6.8	18.1 ± 5.8	0.118
- End of trial	19.9 ± 5.3	21.6 ± 6.1	20.3 ± 4.9	17.5 ± 3.4	
- Adjusted change	1.6 ± 1.0 ^c^	0.4 ± 1.0 ^c^	1.3 ± 0.9 ^c^	0.3 ± 0.9 ^c^	0.636 ^b^
- *p*-value ^a^	0.039	0.900	0.045	0.576	
Protein (g/d)					
- Baseline	68.0 ± 30.1	68.8 ± 18.8	61.4 ± 16.6	60.8 ± 22.2	0.500
- End of trial	72.5 ± 25.5	67.7 ± 18.7	68.8 ± 12.9	61.4 ± 16.6	
- Adjusted change	3.1 ± 3.5 ^c^	−0.3 ± 3.5 ^c^	6.3 ± 3.2 ^c^	2.2 ± 3.2 ^c^	0.690 ^b^
- *p*-value ^a^	0.048	0.654	0.043	0.917	
Carbohydrate (g/d)					
- Baseline	174.8 ± 30.7	194.2 ± 47.1	187.8 ± 42.8	181.3 ± 37.3	0.415
- End of trial	181.3 ± 37.3	193.8 ± 32.5	194.2 ± 47.0	180.1 ± 35.8	
- Adjusted change	8.24 ± 10.8 ^c^	−2.13 ± 10.6 ^c^	8.8 ± 9.9 ^c^	−2.9 ± 9.6 ^c^	0.838 ^b^
- *p*-value ^a^	0.450	0.979	0.655	0.902	
Total fat (g/d)					
- Baseline	43.7 ± 12.5	53.7 ± 13.9	41.7 ± 15.7	46.3 ± 20.3	0.074
- End of trial	53.0 ± 15.5	50.8 ± 19.9	53.7 ± 13.9	43.1 ± 15.0	
- Adjusted change	12.2 ± 4.4 ^c^	−1.3 ± 4.3 ^c^	7.3 ± 4.0 ^c^	−2.2 ± 3.9 ^c^	0.033 ^b^
- *p*-value ^a^	0.051	0.554	0.031	0.453	
Vitamin D (IU/d)					
- Baseline	66.1 ± 46.8	40.4 ± 34.6	41.9 ± 43.5	66.3 ± 39.7	0.052
- End of trial	65.5 ± 44.5	56.0 ± 39.0	60.6 ± 36.4	61.6 ± 46.9	
- Adjusted change	−0.3 ± 8.0 ^c^	10.9 ± 7.9 ^c^	18.0 ± 7.4 ^c^	0.3 ± 7.2 ^c^	0.327 ^b^
- *p*-value ^a^	0.914	0.084	0.058	0.631	
Omega3, EPA (g/d)					
- Baseline	0.037 ± 0.037	0.038 ± 0.035	0.035 ± 0.038	0.038 ± 0.035	0.991
- End of trial	0.042 ± 0.035	0.041 ± 0.036	0.043 ± 0.037	0.037 ± 0.036	
- Adjusted change	0.009 ± 0.010 ^c^	−0.001 ± 0.010 ^c^	0.010 ± 0.009 ^c^	−0.002 ± 0.009 ^c^	0.684 ^b^
- *p*-value ^a^	0.663	0.711	0.436	0.916	
Omega3, DHA (g/d)					
- Baseline	0.085 ± 0.085	0.088 ± 0.078	0.079 ± 0.085	0.087 ± 0.081	0.986
- End of trial	0.092 ± 0.075	0.091 ± 0.074	0.093 ± 0.075	0.085 ± 0.083	
- Adjusted change	0.014 ± 0.020 ^c^	0.001 ± 0.020 ^c^	0.009 ± 0.019 ^c^	−0.001 ± 0.018 ^c^	0.978 ^b^
- *p*-value ^a^	0.737	0.633	0.592	0.939	

ω3; omega-3 group, VitD; vitamin D group, ω3+VitD; vitamin D+omega3 group. EPA; Eicosapentaenoic acid, DHA; Docosahexaenoic acid. ^a^ Within-group differences; *p*-value is based on paired *t*-test. ^b^ Analysis was carried out by covariance (ANCOVA), after adjusting for baseline values, age, income and cancer stage. ^c^ Mean difference represented by adjusted mean and standard error mean.

**Table 4:** Patient-reported adverse events in the different supplementation groups.

Adverse Events	ω3 (*n* = 22)	VitD (*n* = 22)	ω3+VitD (*n* = 22)	Control (*n* = 22)
	*n* (%)			
Nausea	1 (4.5)		1 (4.5)	
Abdominal discomfort	1 (4.5)			
Dry mouth		1 (4.5)		
TOTAL	2 (9.1)	1 (4.5)	1 (4.5)	0

**Table 5:** Comparison of safety biochemical parameters between baseline and end-line of the participants in the different supplementation groups.

Variables	ω3 (*n* = 22)	VitD (*n* = 22)	ω3+VitD (*n* = 22)	Control (*n* = 22)	*p*-Value
	Mean ± SD				
Urea (mg/dL)					
- Baseline	10.55 ± 2.63	10.00 ± 2.62	10.32 ± 3.01	10.86 ± 2.82	0.768
- End of trial	10.91 ± 2.48	11.04 ± 2.70	10.50 ± 2.35	11.00 ± 2.47	
- Adjusted change	0.23 ± 0.79 ^c^	0.75 ± 0.78 ^c^	0.61 ± 0.73 ^c^	0.19 ± 0.70 ^c^	0.752 ^b^
- *p*-value ^a^	0.683	0.115	0.838	0.884	
Creatinine (mg/dL)					
- Baseline	0.86 ± 0.15	0.87 ± 0.18	0.91 ± 0.12	0.85 ± 0.18	0.652
- End of trial	0.90 ± 0.14	0.90 ± 0.14	0.93 ± 0.12	0.92 ± 0.15	
- Adjusted change	0.03 ± 0.04 ^c^	0.01 ± 0.04 ^c^	0.03 ± 0.04 ^c^	0.08 ± 0.04 ^c^	0.291 ^b^
- *p*-value ^a^	0.658	0.888	0.569	0.119	

ω3; omega-3 group, VitD; vitamin D group, ω3+VitD; vitamin D+omega3 group. ^a^ Within-group differences; *p*-value is based on paired *t*-test. ^b^ Analysis was carried out by covariance (ANCOVA), after adjusting for baseline values, age, income and cancer stage. ^c^ Mean difference represented by adjusted mean and standard error mean.

## Data Availability

The dataset used and/or analyzed for the present study is available from the corresponding author on reasonable request. All data underlying the findings of the study are included in this published article.

## References

[1] Sung H., Ferlay J., Siegel R.L., Laversanne M., Soerjomataram I., Jemal A., Bray F. (2021). Global Cancer Statistics 2020: Globocan estimates of incidence and mortality worldwide for 36 cancers in 185 countries. CA Cancer J. Clin..

[2] Siegel R.L., Miller K.D. (2019). Cancer Statistics, 2019. CA Cancer J. Clin..

[3] MOH Palestine (2022). 2022 Health Annual Report Palestine.

[4] de van der Schueren M.A. (2019). Use and effects of oral nutritional supplements in patients with cancer. Nutrition.

[5] Arends J., Bachmann P., Baracos V., Barthelemy N., Bertz H., Bozzetti F., Fearon K., Hütterer E., Isenring E., Kaasa S. (2017). ESPEN guidelines on nutrition in cancer patients. Clin. Nutr..

[6] Adam R., Haileselassie W., Solomon N., Desalegn Y., Tigeneh W., Suga Y., Gebremedhin S. (2023). Nutritional status and quality of life among breast Cancer patients undergoing treatment in Addis Ababa, Ethiopia. BMC Women’s Health.

[7] Polanski J., Chabowski M., Swiatoniowska-Lonc N., Dudek K., Jankowska-Polanska B., Zabierowski J., Mazur G. (2021). Relationship between nutritional status and clinical outcome in patients treated for lung cancer. Nutrients.

[8] Lønbro S., Petersen G.B., Andersen J.R., Johansen J. (2016). Prediction of critical weight loss during radiation treatment in head and neck cancer patients is dependent on BMI. Support. Care Cancer.

[9] Baracos V.E. (2018). Cancer-associated malnutrition. Eur. J. Clin. Nutr..

[10] Nishikawa H., Goto M., Fukunishi S., Asai A., Nishiguchi S., Higuchi K. (2021). Cancer cachexia: Its mechanism and clinical significance. Int. J. Mol. Sci..

[11] Um M.H., Choi M.Y., Lee S.M., Lee I.J., Lee C.G., Park Y.K. (2014). Intensive nutritional counseling improves PG-SGA scores and nutritional symptoms during and after radiotherapy in Korean cancer patients. Support. Care Cancer.

[12] Caccialanza R., Pedrazzoli P., Cereda E., Gavazzi C., Pinto C., Paccagnella A., Beretta G.D., Nardi M., Laviano A., Zagonel V. (2016). Nutritional support in cancer patients: A position paper from the Italian Society of Medical Oncology (AIOM) and the Italian Society of Artificial Nutrition and Metabolism (SINPE). J. Cancer.

[13] Aredes M.A., da Camara A.O., de Paula N.S., Fraga K.Y.D., do Carmo M.D.G.T., Chaves G.V. (2019). Efficacy of ω-3 supplementation on nutritional status, skeletal muscle, and chemoradiotherapy toxicity in cervical cancer patients: A randomized, triple-blind, clinical trial conducted in a middle-income country. Nutrition.

[14] Vrieling A., Seibold P., Johnson T.S., Heinz J., Obi N., Kaaks R., Flesch-Janys D., Chang-Claude J. (2014). Circulating 25-hydroxyvitamin D and postmenopausal breast cancer survival: Influence of tumor characteristics and lifestyle factors?. Int. J. Cancer.

[15] Vaughan-Shaw P.G., O’Sullivan F., Farrington S.M., Theodoratou E., Campbell H., Dunlop M.G., Zgaga L. (2017). The impact of Vitamin D pathway genetic variation and circulating 25-hydroxyVitamin D on cancer outcome: Systematic review and meta-Analysis. Br. J. Cancer.

[16] Khan Q.J., Kimler B.F., Reddy P.S., Sharma P., Klemp J.R., Nydegger J.L., Yeh H.-W., Fabian C.J. (2017). Randomized trial of vitamin D3 to prevent worsening of musculoskeletal symptoms in women with breast cancer receiving adjuvant letrozole. The VITAL trial. Breast Cancer Res. Treat..

[17] Yao S., Kwan M.L., Ergas I.J., Roh J.M., Cheng T.Y.D., Hong C.C., McCann S.E., Tang L., Davis W., Liu S. (2017). Association of serum level of Vitamin D at diagnosis with breast cancer survival a case-cohort analysis in the pathways study. JAMA Oncol..

[18] Carlberg C., Velleuer E. (2022). Vitamin D and the risk for cancer: A molecular analysis. Biochem. Pharmacol..

[19] Muñoz A., Grant W.B. (2022). Vitamin D and cancer: An historical overview of the epidemiology and mechanisms. Nutrients.

[20] Spencer L., Mann C., Metcalfe M., Webb M.B., Pollard C., Spencer D., Berry D., Steward W., Dennison A. (2009). The effect of omega-3 FAs on tumour angiogenesis and their therapeutic potential. Eur. J. Cancer.

[21] Haidari F., Abiri B., Iravani M., Ahmadi-Angali K., Vafa M. (2020). Randomized study of the effect of Vitamin D and omega-3 fatty acids cosupplementation as adjuvant chemotherapy on inflammation and nutritional status in colorectal cancer patients. J. Diet. Suppl..

[22] Bischoff-ferrari H.A., Willett W.C., Manson J.E., Dawson-hughes B. (2022). Combined Vitamin D, omega-3 fatty acids, and a simple home exercise program may reduce cancer risk among active adults aged 70 and older: A randomized clinical trial. Front. Aging.

[23] Story M.J. (2021). Essential sufficiency of zinc, u-3 polyunsaturated fatty acids, vitamin D and magnesium for prevention and treatment of COVID-19, diabetes, cardiovascular diseases, lung diseases and cancer. Biochimie.

[24] Yip C.H., Cazap E., Anderson B.O., Bright K.L., Caleffi M., Cardoso F., Elzawawy A.M., Harford J.B., Krygier G.D., Masood S. (2011). Breast cancer management in middle-resource countries (MRCs): Consensus statement from the Breast Health Global Initiative. Breast.

[25] Rautiainen S., Manson J.E., Lichtenstein A.H., Sesso H.D. (2016). Dietary supplements and disease prevention—A global overview. Nat. Rev. Endocrinol..

[26] Qiu Y., You J., Wang K., Cao Y., Hu Y., Zhang H., Fu R., Sun Y., Chen H., Yuan L. (2020). Effect of whole-course nutrition management on patients with esophageal cancer undergoing concurrent chemoradiotherapy: A randomized control trial. Nutrition.

[27] Craig C.L., Marshall A.L., Sjöström M., Bauman A.E., Booth M.L., Ainsworth B.E., Pratt M., Ekelund U., Yngve A., Sallis J.F. (2003). International physical activity questionnaire: 12-Country reliability and validity. Med. Sci. Sports Exerc..

[28] Ottery F.D., Wagner K., Dixon S. (2016). Integrating routine nutritional screenings for cancer patients at the point of care: Pilot testing a novel care planning system plus certified professional training. Postgrad. Inst. Med. Carevive Syst..

[29] Ottery F.D. (2001). Physical Examination from a Nutritional Standpoint. www.pt-global.org.

[30] Ottery F.D. (1996). Definition of standardized nutritional assessment and interventional pathways in oncology. Nutrition.

[31] IBM Corp (2020). IBM SPSS Statistics for Windows, Version 27.0.

[32] Bering T., Mauricio S.F., da Silva J.B., Davisson Correia M.I.T. (2015). Nutritional and metabolic status of breast cancer women. Nutr. Hosp..

[33] Wong T.X., Wong W.X., Chen S.T., Ong S.H., Shyam S., Ahmed N., Hamdan K.H., Awang R.R., Ibrahim M.R., Palayan K. (2022). Effects of perioperative oral nutrition supplementation in malaysian patients undergoing elective surgery for breast and colorectal cancers—A randomised controlled trial. Nutrients.

[34] Lee K., Kruper L., Dieli-Conwright C.M., Mortimer J.E. (2019). The impact of obesity on breast cancer diagnosis and treatment. Curr. Oncol. Rep..

[35] Arends J., Baracos V., Bertz H., Bozzetti F., Calder P.C., Deutz N.E.P., Erickson N., Laviano A., Lisanti M.P., Lobo D.N. (2017). ESPEN expert group recommendations for action against cancer-related malnutrition. Clin. Nutr..

[36] Muscaritoli M., Arends J., Bachmann P., Baracos V., Barthelemy N., Bertz H., Bozzetti F., Hütterer E., Isenring E., Kaasa S. (2021). ESPEN practical guideline: Clinical nutrition in cancer. Clin. Nutr..

[37] Chaves M.R., Boléo-Tomé C., Monteiro-Grillo I., Camilo M., Ravasco P. (2010). The diversity of nutritional status in cancer: New insights. Oncologist.

[38] Nasrah R., Kanbalian M., Van Der Borch C., Swinton N., Wing S., Jagoe R.T. (2018). Defining the role of dietary intake in determining weight change in patients with cancer cachexia. Clin. Nutr..

[39] Kocarnik J.M., Hua X., Hardikar S., Robinson J., Lindor N.M., Win A.K., Hopper J.L., Figueiredo J.C., Potter J.D., Campbell P.T. (2017). Long-term weight loss after colorectal cancer diagnosis is associated with lower survival: The Colon Cancer Family Registry. Cancer.

[40] Cheng M., Zhang S., Ning C., Huo Q. (2021). Omega-3 Fatty acids supplementation improve nutritional status and inflammatory response in patients with lung cancer: A randomized clinical trial. Front. Nutr..

[41] Feijó P.M., Rodrigues V.D., Viana M.S., dos Santos M.P., Abdelhay E., Viola J.P., de Pinho N.B., Martucci R.B. (2019). Effects of ω-3 supplementation on the nutritional status, immune, and in fl ammatory pro fi les of gastric cancer patients: A randomized controlled trial. Nutrition.

[42] Levitt D.G., Levitt M.D. (2016). Human serum albumin homeostasis: A new look at the roles of synthesis, catabolism, renal and gastrointestinal excretion, and the clinical value of serum albumin measurements. Int. J. Gen. Med..

[43] Eckart A., Struja T., Kutz A., Baumgartner A., Baumgartner T., Zurfluh S., Neeser O., Huber A., Stanga Z., Mueller B. (2020). Relationship of nutritional status, inflammation, and serum albumin levels during acute illness: A prospective study. Am. J. Med..

[44] Sim E., Kim J.M., Lee S.M., Chung M.J., Song S.Y., Kim E.S., Chun H.J., Sung M.K. (2022). The Effect of omega-3 enriched oral nutrition supplement on nutritional indices and quality of life in gastrointestinal cancer patients: A randomized clinical trial. Asian Pac. J. Cancer Prev..

[45] Lee J.L.C., Leong L.P., Lim S.L. (2016). Nutrition intervention approaches to reduce malnutrition in oncology patients: A systematic review. Support. Care Cancer.

[46] Freitas R.D.S., Campos M.M. (2019). Protective effects of omega-3 fatty acids in cancer-related complications. Nutrients.

[47] Klek S. (2016). Omega-3 fatty acids in modern parenteral nutrition: A review of the current evidence. J. Clin. Med..

[48] Mocellin M.C., de Quadros Camargo C., de Souza Fabre M.E., de Moraes Trindade E.B.S. (2017). Fish oil effects on quality of life, body weight and free fat mass change in gastrointestinal cancer patients undergoing chemotherapy: A triple blind, randomized clinical trial. J. Funct. Foods.

[49] Koole J.L., Bours M.J., van Roekel E.H., Breedveld-Peters J.J., van Duijnhoven F.J., van Den Ouweland J., Breukink S.O., Janssen-Heijnen M.L., Keulen E.T., Weijenberg M.P. (2020). Higher serum vitamin d concentrations are longitudinally associated with better global quality of life and less fatigue in colorectal cancer survivors up to 2 years after treatment. Cancer Epidemiol. Biomark. Prev..

[50] Martínez-Alonso M., Dusso A., Ariza G., Nabal M. (2016). Vitamin D deficiency and its association with fatigue and quality of life in advanced cancer patients under palliative care: A cross-sectional study. Palliat. Med..

[51] Lewis C., Xun P., He K. (2016). Vitamin D supplementation and quality of life following diagnosis in stage II colorectal cancer patients: A 24-month prospective study. Support. Care Cancer.

[52] Lee S.M., Son Y.K., Kim S.E., An W.S. (2015). The effects of omega-3 fatty acid on vitamin d activation in hemodialysis patients: A pilot study. Mar. Drugs.

[53] Alhabeeb H., Kord-Varkaneh H., Tan S.C., Găman M.A., Otayf B.Y., Qadri A.A., Alomar O., Salem H., Al-Badawi I.A., Abu-Zaid A. (2022). The influence of omega-3 supplementation on vitamin D levels in humans: A systematic review and dose-response meta-analysis of randomized controlled trials. Crit. Rev. Food Sci. Nutr..

[54] Patrick R.P., Ames B.N. (2015). Vitamin D and the omega-3 fatty acids control serotonin synthesis and action, part 2: Relevance for ADHD, bipolar disorder, schizophrenia, and impulsive behavior. FASEB J..

[55] Partan R.U., Hidayat R., Saputra N., Rahmayani F., Prapto H., Yudha T.W. (2019). Seluang fish (*Rasbora* spp.) oil decreases inflammatory cytokines via increasing vitamin d level in systemic lupus erythematosus. Open Access Maced. J. Med. Sci..

[56] Peppone L.J., Huston A.J., Reid M.E., Rosier R.N., Zakharia Y., Trump D.L., Mustian K.M., Janelsins M.C., Purnell J.Q., Morrow G.R. (2011). The effect of various vitamin D supplementation regimens in breast cancer patients. Breast Cancer Res. Treat..

[57] Sánchez-Lara K., Turcott J.G., Juárez-Hernández E., Nuñez-Valencia C., Villanueva G., Guevara P., De la Torre-Vallejo M., Mohar A., Arrieta O. (2014). Effects of an oral nutritional supplement containing eicosapentaenoic acid on nutritional and clinical outcomes in patients with advanced non-small cell lung cancer: Randomised trial. Clin. Nutr..

[58] Lenz J.S., Tintle N., Kerlikowsky F., Badrasawi M., Zahdeh R., Qasrawi R., Hahn A., Schuchardt J.P. (2023). Assessment of the vitamin D status and its determinants in young healthy students from Palestine. J. Nutr. Sci..

